# Parametrizing the genioplasty: a biomechanical virtual study on soft tissue behavior

**DOI:** 10.1007/s11548-021-02489-9

**Published:** 2021-09-17

**Authors:** F. Ruggiero, G. Badiali, M. Bevini, C. Marchetti, J. Ong, F. Bolognesi, S. Schievano, D. Dunaway, A. Bianchi, A. Borghi

**Affiliations:** 1 DIBINEM Alma Mater Studiorum Bologna, Bologna, Italy; 2 Craniofacial Group, UCL Great Ormond Street Institute of Child Health-The Zayed Centre for Research, 20 Guilford Street, London, WC1N 1DZ UK; 3 Division of Craniofacial and Plastic Surgery, Great Ormond Street Hospital for Children, London, UK; 4 Oral and Maxillo-facial Unit, University of Catania, Catania, Italy

**Keywords:** Genioplasty, Orthognathic surgery, Finite element method, Numerical modeling

## Abstract

**Purpose:**

Sliding genioplasty is used to surgically correct a retruded or misaligned chin: in this procedure, an osteotomy is performed and the bony segment is repositioned. In this study we investigate the effect of surgical parameters (bony segment movement, osteotomy design) on postop soft tissue changes in a patient cohort.

**Methods:**

Seven patients were retrospectively recruited. Cone beam computed tomography data were obtained and soft tissue and bone shape reconstructions were performed. 3D models were created and surgical cuts were replicated according to postop scans. Each model was imported in ANSYS 2019R1 (Ansys Inc, USA) for simulation: the effect of variation in osteotomy plane as well as extent of bony segment movement were assessed by means of design of experiment: surgical parameters were varied in a surgically acceptable range and the soft tissue predictions were evaluated as displacement output of five craniometric landmarks.

**Results:**

Simulation results show the overall changes of the lower third of the face are sensitive to changes in horizontal and vertical displacement of the bony segment as well as segment rotation. No significant changes in the soft tissue response were to attribute to the osteotomy design.

**Conclusions:**

Our results are consistent with experimental findings reported in the literature: when planning genioplasty in orthognathic surgery, particular focus on the segment movement (horizontal translation, vertical translation and rotation), rather than on the design of the osteotomy itself, should be considered.

## Introduction

Repositioning the chin during orthognathic surgery, also called genioplasty or mentoplasty, is a common procedure both for cosmetic and functional purposes [1, 2], where the surgeon cuts a portion of the chin bone and repositions it surgically. The overall harmony of the face is due to the contribution of the balance of its different aesthetic units [3], in which the chin plays a key role [4–6]. Even to an untrained eye, the lower third of the face is the most important area when evaluating aesthetics and an imbalance in this structure, in terms of shape, size and position, can have considerable results on the final outcome [7, 8].

Several techniques have been described in the literature since its first descriptions [9], aiming to address different chin aspects: sliding advancement genioplasty is the most common, but also setback genioplasty [10], impaction or intrusion genioplasty [11], vertical height augmentation genioplasty [12], and narrowing or widening mentoplasty procedures [13, 14].

The predictability of osteotomy and bony repositioning results on the soft tissue is still controversial. A wide literature can be found on soft tissue/skeletal tissue displacement ratio in chin sliding genioplasty [1, 3, 7, 8, 15]. A more specific knowledge of skin surface response after genioplasty is advisable in order to provide a better outcome [3, 16]. Outcome predictions have been carried out using either standard bi-dimensional cephalometric analysis or three-dimensional (3D) commercial software. Our 3D method of choice is the finite element method (FEM), which has already been described in the literature as a viable and accurate method for predicting soft tissue changes in orthognathic surgery [17–19].

We hereby present a virtual retrospective study of the shape change of the lower third of the face following sliding genioplasty, according to the choice of surgical parameters such as osteotomy position and extent of bony segment movement.

## Methodology

Seven patients (one male and six females) were selected within a cohort of patients admitted to the Maxillofacial Unit of Sant’Orsola University Hospital (Bologna), for orthognathic surgery with sliding genioplasty. All the patients included received 3D CBCT preoperatively and a postoperative CBCT at follow-up. All individuals presented with no skin defects and no diagnosis of craniofacial syndrome. All procedures performed in studies involving human participants were in accordance with the ethical standards of the institutional and/or national research committee and with the 1964 Helsinki declaration and its later amendments or comparable ethical standards. For this type of retrospective study formal consent is not required. Following general anesthesia, each patient underwent sliding genioplasty, where the chin bone was separated from the rest of the jaw and repositioned along a horizontal vector (either in anterior or posterior direction—Table 1), a vertical vector (either in cranial or caudal direction—Table 1) and rotated (either clockwise or anticlockwise—Table 1). For each patient we retained preoperative and postoperative (six months after surgery) CBCT scans, which were taken with the same machine (NewTom 3000). Table 1 reports a summary of the patients at the age of surgery.

**Table 1:** Summary of the patients recruited for this study and the surgical parameters used in the finite element model to replicate the genioplasty segment repositioning: horizontal displacement UH (a: anterior direction, p: posterior direction), vertical displacement UV (cra: cranial direction, cau: caudal direction), rotation ϑ (c: clockwise, cc: counterclockwise), osteotomy height H, osteotomy angle α

Patient	Sex	Age at procedure (years)	*UH*^*S*^ (mm)	*UV*^*S*^ (mm)	*ϑ*^*S*^ (°)	*H*^*S*^ (mm)	*α*^*S*^ (°)
P1	F	16	2 (a)	0	10 (cc)	14.0	13.0
P2	F	19	4 (a)	1 (cau)	11.7 (cc)	20.3	10.4
P3	M	24	1 (p)	1 (cra)	8 (c)	24.0	5.7
P4	F	17	0.5 (a)	0.5 (cau)	8.4 (cc)	22.0	15.0
P5	F	32	2 (a)	2 (cra)	16 (cc)	18.0	19.0
P6	F	35	2 (a)	4 (cau)	8 (cc)	26.5	3.0
P7	F	39	1 (a)	1.5 (cau)	5 (cc)	26.0	2.0

**Fig. 1:**
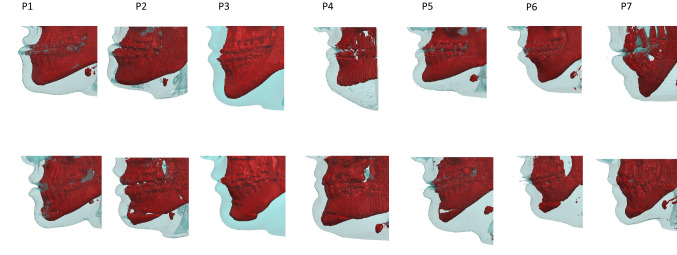
Summary of all the patients included in the study with preoperative (top row) and postoperative (bottom row) 3D reconstruction of hard (red) and soft (transparent blue) tissue

**Fig. 2:**
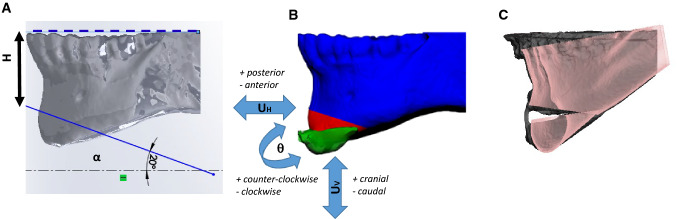
**a** Parametrization of the genioplasty osteotomy: *α* is the angle, *H* is the distance of the osteotomy plane from the lower incisal plane **b** Parameters of bony segment repositioning: horizontal translation (*U*_*H*_, negative in the anterior direction, positive in the posterior direction), vertical translation (*U*_*V*_, negative in the caudal direction, positive in the cranial direction) and pitch rotation (*ϑ*, positive when counterclockwise, negative when clockwise when viewed from the left side). **C** Bony segment repositioning (using the parameter set *H*^*S*^, *α*^*S*^, *UH*^*S*^, *UV*^*S*^, *ϑ*^*S*^) to match the postop mandible-chin relative position (in gray the postoperative chin shape extracted from CBCT; in transparent pink the simulated surgical result for a representative patient)

To model the surgery, DICOM data were exported and imported in SCANIP® (Synopsis, Mountain View, CA): hard and soft tissues were segmented using gray-value thresholding (Fig. 1). Table 1 shows a summary of the population considered. Each preoperative patient anatomy was cut at the incisor level superiorly and midway through the mandible rami posteriorly. NURBS (non-uniform-rational-b-spline) surfaces were created for both soft and hard tissue and imported into Solidworks 2018 (Dassault Systémes, France). A plane was created to split the mandible into genioplasty segment and main body. Such plane was parametrized in terms of angle with the horizontal direction (*α*) and distance between the lower incisal plane and the osteotomy plane (H—Fig. 2A). The assembly soft tissue—hard tissue was imported into ANSYS 2019R1 (USA). Material properties for mandible and soft tissue were retrieved from the literature and summarized in Table 2 [19]. The selected patients belong to a historical cohort of individuals who underwent orthognathic surgery and genioplasty segment repositioning was carried out without the assistance of a navigation tracking system. The plane for the osteotomy, as well as the movement, were replicated by superimposing the preoperative and postoperative mandible using iterative closest point algorithm in the regions of the ramus and body (see Fig. 2C). Following virtual osteotomy, the surgical procedure of genioplasty segment repositioning was simulated by applying a rigid roto-translation to the top surface of the segment, defined by horizontal translation (*UH*, assumed positive in the posterior direction), a vertical translation (*UV*, assumed positive in the cranial direction) and a pitch rotation (ϑ, assumed positive when counterclockwise—Fig. 2B). Yaw and roll rotation were not considered. The osteotomy (*H*, *α*) and repositioning (*UH*, *UV*, *ϑ*) parameters were fine-tuned by trial and error to find the exact combination of values replicating the surgical osteotomy and repositioning (*H*^*s*^, *α*^*s*^, *UH*^*s*^, *UV*^*s*^, ϑ^*s*^) for each single patient (Fig. 2C, Table 1).

**Table 2:** Biomechanical properties for soft and hard tissue

	Young’s modulus *E* (MPa)	Poisson’s ratio *n*	From
Bone	7930	0.3	[42]
Soft tissue	1	0.49	[19]

Each set of hard and soft tissue was discretized using tetrahedral elements. The superior and posterior surfaces of the 3D model were fixed to mimic tethering of the other tissues. Surgical repositioning of the genioplasty segment was simulated for each patient using the surgical parameter combination (*H*^*s*^, *α*^*s*^, *UH*^*s*^, *UV*^*s*^, *ϑ*^*s*^). Simulated postoperative soft tissue surface was extracted and compared with the postoperative CBCT soft tissue reconstruction for validation. The superimposition previously used to replicate the osteotomy plane was used for the comparison of the FE predicted postop soft tissue (which was in the same reference framework as the preoperative mandible) with the segmented postoperative soft tissue (which was in the same reference framework as the postop mandible, which had been previously been registered to the pre-operative anatomy).

**Fig. 3:**
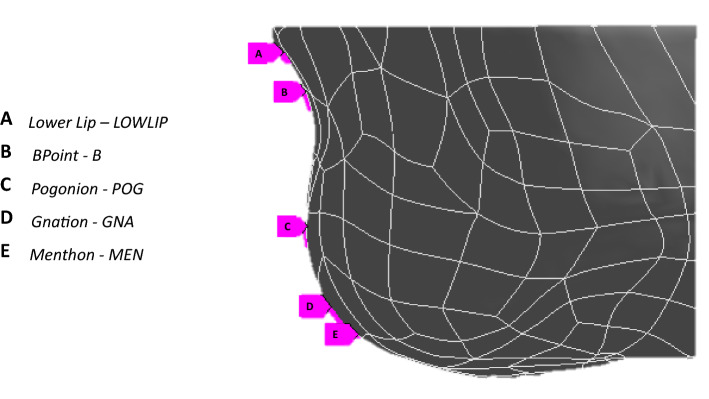
Cephalometric landmarks used in this study

On each patient model, 5 cephalometric landmark points were identified (Fig. 3). Table 4 reports the definition of each cephalometric landmark. Design of experiments (DoE, a standard engineering technique to minimize the number of experiments necessary to characterize parametric sensitivity of a mechanical system) was performed to investigate the effect of variation in osteotomy parameters and extent of surgical repositioning on the vertical and horizontal displacement of the 5 cephalometric landmark points. Tables 3 and 4 summarize the input and output variables of each model. For each patient model, 27 simulations for a total of 189 simulations for the whole population) were performed, where input parameters were varied in the predefined range, and output variables were recorded. Each simulation (“*design point*”) had a different set of input parameters. The sensitivity of each output variables to the variation in input (defined as the rate of output change versus change in input) was extracted from the results and compared throughout the population. The sensitivity of each output variables was averaged throughout the cohort and statistical difference analyzed. Local sensitivity charts were created: each chart bar shows the effect of continuous input parameters on output parameters. The maximum variation in horizontal and vertical displacement for each cephalometric landmark throughout the whole set of simulations was analyzed, in order to assess which surgical parameters have the highest influence on the results of the genioplasty. Statistical differences were assessed using the Wilcoxon-rank test (*p* < 0.05 was assumed significant).

**Table 3:** On the left column the input variables considered (*H*: height of the osteotomy, *α*: osteotomy angle, *U*_*H*_: horizontal translation of the bone fragment, *U*_*v*_: vertical translation of the bone fragment, *ϑ*: rotation of the bone fragment), on the right column the range of variation for each variable used in the DoE

Input variable	Range
*H*	[*H*^S ^− 1 mm, H^S^ + 1 mm]
*α*	[*α*^*S* ^− 2°, *α*^*S*^ + 2°]
*U*_*H*_	[*U*_*H*_^*S* ^− 1 mm, *U*_*H*_^*S*^ + 1 mm]
*U*_*V*_	[*U*_*V*_^*S* ^− 1 mm, *U*_*V*_^*S*^ + 1 mm]
*θ*	[*ϑ*^S ^− 2°, *ϑ*^*S*^ + 2°]

**Table 4:** The cephalometric landmarks considered (on the left column), their definition (in the middle column), the output variables considered for each point (in the right column)

Cephalometry landmark	Definition [43]	Output variables
LOW LIP	Midpoint of the border of the lower lip	LOWLIP_H_, LOWLIP_V_
B POINT	The innermost on the mandible contour	*B*_*H*_, *B*_*V*_
POGONION	The most anterior point of the chin	POG_H_, POG_V_
GNATION	The lowest, most anterior midline point on the symphysis of the mandible	GNA_H_, GNA_V_
MENTHON	The most inferior point on the mandibular symphysis	MEN_H_, MEN_V_

## Results

**Fig. 4:**
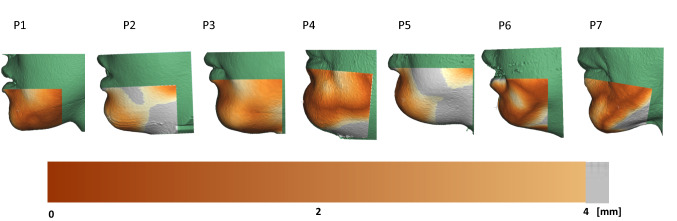
Heat maps showing the surface difference between the simulated soft tissue change and the postoperative reconstruction from CBCT

Genioplasty surgery was initially simulated in all seven patients using the combination of values replicating the surgical osteotomy and repositioning (*H*^*s*^, *α*^*s*^, *UH*^*s*^, *UV*^*s*^, *ϑ*^*s*^), in order to validate the overall method. Figure 4 shows heat maps relative to the surface distance between the simulated outcome and the postoperative lower face model extracted from CBCT.

**Fig. 5:**
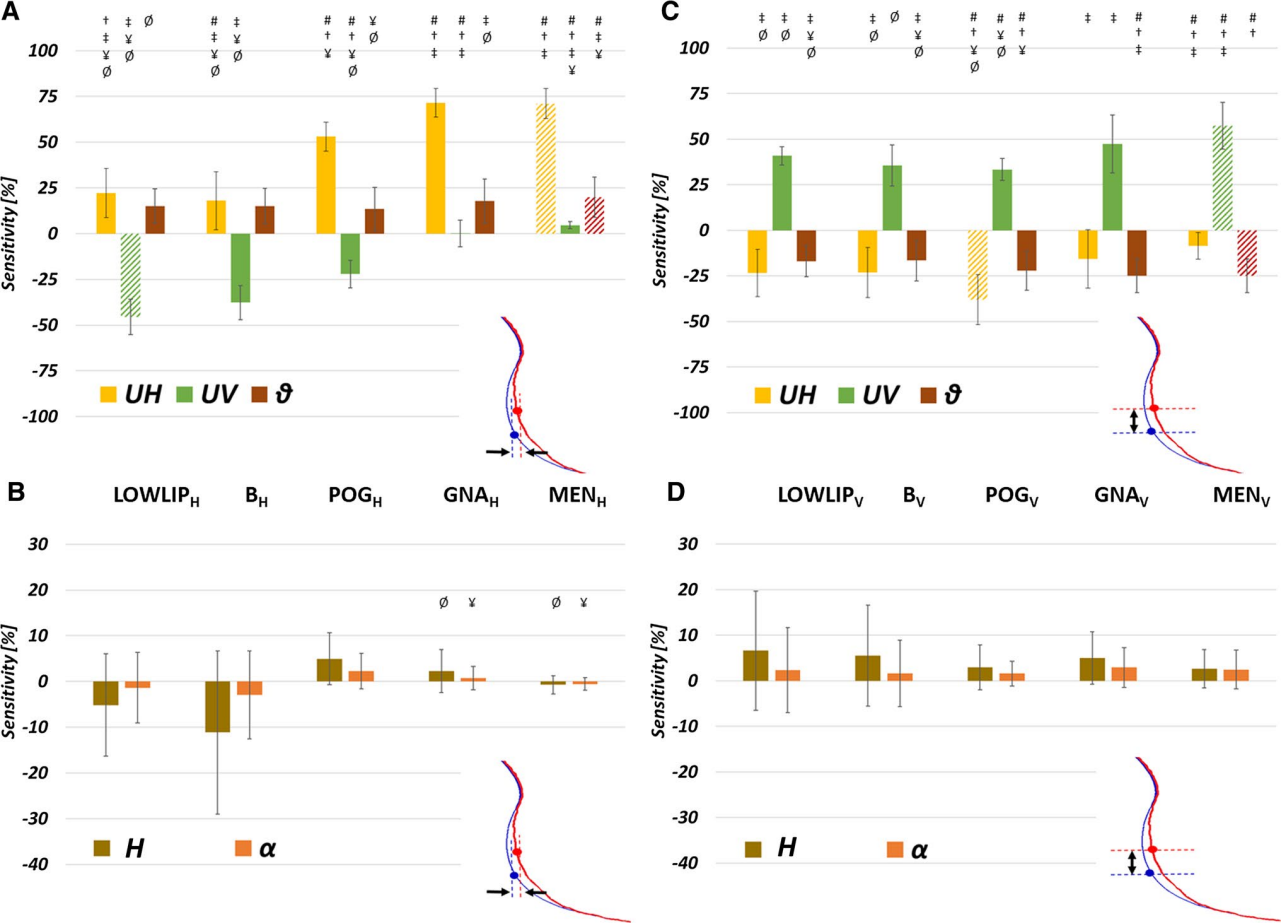
Sensitivity for each cephalometric landmark considered on the horizontal (**a**, **b**) and vertical (**c**, **d**) displacement, to the surgical parameters (*UH*, *UV*, *ϑ* on the top row; *H*, *α* on the bottom row). The largest (positive or negative) sensitivities recorded for each surgical parameter are highlighted with oblique bars. Symbols show statistical differences (*p* < 0.05): # means statistically different from LOWLIP, † means statistically different from B, ‡ means statistically different from POG, ¥ means statistically different from GNA, Ø means statistically different from MEN

The effect of changing surgical parameters in terms of osteotomy position (*H*, *α*) and extent of repositioning (*UH*, *UV*, *ϑ*) on the post-procedural position of the cephalometric landmarks analyzed was assessed. Figure 5 shows the sensitivity of the horizontal (Fig. 5a, b) and vertical (Fig. 5c, d) displacement of the cephalometric landmarks on the surgical parameters (symbols report statistical differences). Table 5 reports the numerical values of all the sensitivities which are compared on Fig. 5. Oblique bars in Fig. 5a, c highlight the cephalometric landmarks that are mostly affected by the segment movements on the horizontal and vertical directions.

**Table 5:** Sensitivity of each landmark’s horizontal (H) and vertical (V) displacement according to the surgical parameters

	UH	UV	ϑ	H	α
LOWLIP_H_	22.23 ± 13.46	− 45.5 ± 9.72	15.17 ± 9.42	− 5.17 ± 11.19	− 1.38 ± 7.71
LOWLIP_V_	− 23.37 ± 12.89	40.89 ± 5.06	− 16.89 ± 8.58	6.64 ± 13.07	2.35 ± 9.32
B_H_	18.06 ± 15.80	− 37.65 ± 9.29	15.07 ± 9.63	− 11.15 ± 17.85	− 2.95 ± 9.62
B_V_	− 23.2 ± 13.73	35.64 ± 11.28	− 16.51 ± 11.12	5.52 ± 11.09	1.60 ± 7.27
POG_H_	53.01 ± 7.83	− 22.04 ± 7.53	13.44 ± 11.82	4.97 ± 5.67	2.30 ± 3.87
POG_V_	− 38.07 ± 13.7	33.38 ± 6.08	− 21.98 ± 10.77	2.93 ± 4.92	1.59 ± 2.74
GNA_H_	71.57 ± 7.82	0.06 ± 7.36	17.81 ± 12.06	2.23 ± 4.70	0.72 ± 2.56
GNA_V_	− 15.61 ± 16.07	47.43 ± 15.89	− 24.89 ± 9.32	4.98 ± 5.74	2.91 ± 4.31
MEN_H_	71.12 ± 8.11	4.62 ± 1.92	19.80 ± 10.9	− 0.70 ± 1.90	− 0.50 ± 1.30
MEN_V_	− 8.50 ± 7.32	57.37 ± 12.86	− 25.03 ± 9.22	2.67 ± 4.21	2.46 ± 4.26

**Fig. 6:**
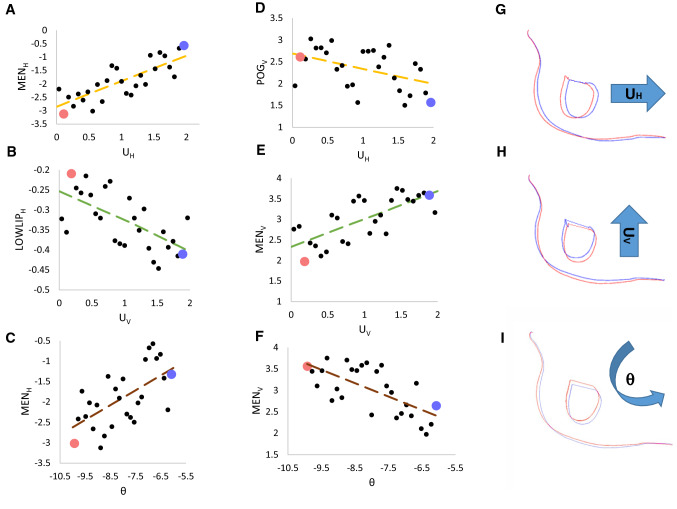
For a representative patient, relationship between genioplasty segment movement (UH, UV, *θ*) and the horizontal (**a–c**) and vertical (**d–f**) displacement of the most affected cephalometric landmarks (highlighted in Fig. 5 with oblique bars) throughout all design points simulated. Two design points having high (blue) and low (red) input values were selected for each input parameter and the simulated postoperative chin shape is shown on the left (**g–i**) with matching silhouette color; the arrows highlight the positive direction of the displacement (posterior for the horizontal displacement, cranial for the vertical displacement) and rotations (counterclockwise)

For a representative patient, Fig. 6a–c shows the largest sensitivity in variations encountered in horizontal displacement when varying the genioplasty segment movement parameters (*U*_*H*_, *U*_*V*_ and *ϑ*), while Fig. 6d–f shows the highest sensitivity in vertical displacement when varying these parameters. For each graph pair, two design points having high and low input parameter values (depicted in red and blue, respectively) were selected and the relative simulated postsurgical shapes were extracted: a sagittal cut shows the change in chin aspect (color match the relative design point).

**Fig. 7:**
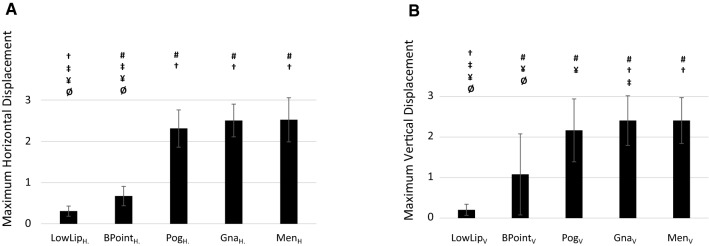
Histogram showing peak horizontal (**a**) and vertical (**b**) displacement achieved throughout all the simulations for each craniometric landmark. Symbols show statistical differences (*p* < 0.05): # means statistically different from LOWLIP, † means statistically different from B, ‡ means statistically different from POG, ¥ means statistically different from GNA, Ø means statistically different from MEN

The maximum calculated horizontal and vertical displacement reported by the cephalometric landmarks throughout all of the design points (reported in Fig. 7a, b) were extracted. Larger horizontal displacements (*p* < 0.05, Fig. 7a) were reported for MEN (MEN_H_ = 2.52 ± 0.54), GNA (GNA_H_ = 2.51 ± 0.40) and POG (POG_H_ = 2.31 ± 0.45) compared to LOWLIP (LOWLIP_H_ = 0.31 ± 0.12), B (B_H_ = 0.67 ± 0.23); similarly, larger vertical displacements (*p* < 0.05, Fig. 7b) were reported for MEN (MEN_V_ =), GNA (GNA_V_ = 2.41 ± 0.61) and POG (POG_V_ = 2.17 ± 0.78) compared to LOWLIP (LOWLIP_V_ = 0.20 ± 0.14), BPOINT (B_V_ = 1.08 ± 1.00). LOWLIP experienced a lower vertical and horizontal displacement than the B (*p* < 0.05) and POG showed a lower vertical displacement than the GNA (*p* = 0.047).

## Discussion

Genioplasty plays a key role in the overall balancing of the profile and therefore it is generally performed for cosmetic purposes. In this study we mainly focused on the most common technique which is the sliding genioplasty. Due to the heavy aesthetic implications and the importance of the lower third in the total facial harmony, planning this kind of surgery is of paramount importance [7]. Although for many years bi-dimensional cephalometric studies have been the gold standard for surgical planning, over the last decade surgeons have taken advantage of more recent 3D planning techniques such as FEM [20, 21]. FEM allows for solution of complex physical problem while taking into account individual tissue mechanical properties. The use of DoE allowed the analysis of the effect of the change in input surgical parameters (osteotomy location and extent of bony segment repositioning) on the change in shape of the lower third of the face.

In this study we evaluated the changes of the chin main cephalometric landmarks in 7 patients who underwent genioplasty. To validate our method, a first group of simulations was carried out using a set of parameters which replicated the actual surgical procedure. The simulated postoperative chin shape showed good matching with the shape extracted from the CBCT acquired postoperatively. The bony repositioning was successfully simulated (although minor chin reshaping achieved by filing the bony segment could not be reproduced) and the surface discrepancy in the area near the chin was below 2 mm (as shown by Fig. 4), which is considered an acceptability threshold in cranio-maxillofacial surgery planning [22]. Small mismatch below the chin and on the cheeks was shown in some of the patients, this possibly due to factors related to the surgery (mandibular repositioning along with genioplasty was performed in some patients—see P3) or postoperative loss/gain of weight.

The DoE results showed that extent of bony segment repositioning (in terms of displacement and rotation of the bony segment) has a more pronounced effect on the final shape of the chin, than the position and angle of the osteotomy. The simulations showed that UH has a consistently positive effect on the horizontal displacement of all craniometrics points while it has a negative effect on the vertical displacement of all points: this means that a more pronounced anterior advancement will both advance and pull upward the chin surface. The opposite applies to *UV*: negative effect is visible on the horizontal displacement of the cephalometric landmarks, while positive effect is visible on the vertical displacement. This reflects the structural continuity of the chin tissue, whose effect has been reported in the literature: Van Sickels et al. [23] reported that soft tissue thickness decreases with chin advancement.

According to these findings the B, the POG and the LOWLIP—responsible for this aesthetic unit—respond to the horizontal displacement of the segment, with a visible effect in craniocaudal direction. Vertical advancement/setback seems has a major influence on POG, GNA and MEN on the sagittal plane: this is consistent with reports from the literature which state that GNA and MEN respond with a major change in craniocaudal sense to vertical displacement, with a positive correlation [8, 24–27]. It is well known that—in case of advancement genioplasty—the labiomental fold (crease) increases [28]: a higher sensitivity of the distal craniometrics points (GNA, MEN) compared to the proximal points (LOWLIP, B) shows that a more pronounced bony segment movement accentuates this feature.

The horizontal displacement of the bony segment, on the other side, influences the position of the low lip on the sagittal plane: therefore, the more forward moves the chin on the axial plane, the more the lower lip moves on the sagittal plane, therefore having an aesthetic influence on top of the more obvious functional one (lip competence). Rotation caused a relatively constant positive variation on the horizontal displacement and a negative variation which increased distally (from LOWLIP to MEN).

Variation in H and α had inconsistent effect throughout the population: as both variables were varied from the original surgical configuration, differences in the initial surgical strategy (clockwise vs anticlockwise rotation, distal vs proximal osteotomy) had an impact on the range of retrievable outputs. A study by Möhlhenrich et al. [8] reported an effect of varying osteotomy angle in sliding genioplasty, however a larger range of segment displacement (0 to 10 mm) and rotation (± 5°) was considered and the results show that—when the same extent of chin repositioning is considered—the effect of changing the rotation is low although consistent. The author themselves report that only in the most extreme case, visible effect was present on the inferior soft tissue when varying the osteotomy angle.

In this work, the authors produced a numerical model to assess the sensitivity of genioplasty surgical outcome to surgical parameters (osteotomy position and segment repositioning). This is a simplified model which only accounts for the presence of soft and bony tissues without subdividing the former into its main components (fibers, fat, glands, mucosa and three layers of skin). Although the homogeneous tissue assumption has been used in other studies in the past where orthognatic surgery was simulated [19, 29] and it is the underlying assumption of preoperative planning commercial softwares such as Materialize Proplan [8, 30], a more anatomically accurate representation of the underlying tissues [31–35] would provide a better representation of the elastic response of the lower third soft tissue. Current work in our group is aiming at combining different imaging modalities to overcome the current simplification and provide a more detailed representation of the patient-specific face anatomy [36]. Payan et al. [34] proposed a method for calibrating soft tissue material properties using patient-specific measurements gathered using an aspiration device: our cohort was retrospectively recruited and therefore patient-specific assessment of tissue properties would not be possible. Model validation (reported in Fig. 4) shows that the chin area change is correctly predicted in all patients and therefore it is reasonable to assume that the following sensitivity analysis, which focuses on cephalometric points located between the lower lip and the chin, is valid and accurate.

This group of patients received CBCT as a standard imaging method for surgical preoperative planning adopted on our center. CT has been reported to provide better hard tissue magnification and is ideal for hard tissue segmentation using appropriate Hounsfield unit (HU) range. However, it has been reported that there is a strong correlation between gray scales of CBCT and HU of CT scan [37] and most works on orthognatic surgery planning have been carried out using clinically available CBCT [17, 18]. Furthermore, several study compared linear measurements in vitro [38, 39] and ex vivo [40] finding CBCT and standard multi-slice CT equally accurate in replicating anatomical dimensions.

The method used for replicating the surgical scenario was adapted from Knoops et al. [19, 41] who carried out a retrospective simulation of orthognathic surgery in a retrospective patient group. To replicate the osteotomy, rigid ICP (implemented in meshmixer) was used to register the preoperative and postoperative mandible in the region of the ramus and body (which are not affected by the genioplasty repositioning). A total of 100 iterations with an error tolerance of 0.01 mm was used. The most common methodology for registering preoperative and postoperative scans used in the literature is to perform a registration based on the skull base. Such method is suitable for assessing changes in the midface (whose position is fixed with the skull base), however it was not applicable to all patients, either because of minor mismatch in the mandibular position (due to autorotation when genioplasty occurred in conjunction with maxillary repositioning) or due to the absence of the skull base in the pre or postoperative scan.

In conclusion, the overall changes of the lower third of the face are sensitive to changes in the amount of advancement and set back as well as vertical displacement of the bony segment. Therefore, when planning genioplasty in orthognathic surgery, particular focus on the horizontal and vertical movements of the segment, rather than the design of the osteotomy itself, should be considered. When the aesthetic target of the surgery is to modify the labiomental fold, particular attention should be paid to the frontal and vertical shifting of the bony segment, which has a differential effect on proximal and distal craniometrics landmarks.
